# Recommendations for promoting international multi‐site clinical trials—from a viewpoint of ethics review

**DOI:** 10.1111/dewb.12245

**Published:** 2019-09-12

**Authors:** Haruka Nakada, Suzanne Hasthorpe, Carel IJsselmuiden, Francis Kombe, Marieme Ba, Mihaela Matei, Kenichi Nakamura, Nobuko Ushirozawa, Yasuhiro Fujiwara, Shimon Tashiro

The early 21^st^ century has seen a growing trend to perform large‐scale clinical trials, in general, and across national borders, in particular.1Atal, I., Trinquart, L., Porcher, R., Ravaud, P. (2015). Differential globalization of industry‐ and non‐industry‐sponsored clinical trials. PLoS One. 10(12), 1‐17. https://doi.org/10.1371/journal.pone.0145122.
^,^ 2Viergever, R.F., Li, K. (2015). Trends in global clinical trial registration: An analysis of numbers of registered clinical trials in different parts of the world from 2004 to 2013. BMJ Open. 5(9). https://doi.org/10.1136/bmjopen-2015-008932. Major reasons for this trend include the collective promotion of global health and the need to rapidly and effectively respond to threats to human health worldwide. The latest epidemic of Ebola Virus Disease (EVD) showed us that the systems put in place for the development of international clinical research were not ready to face the challenge of controlling that deadly disease.3Keusch, G., McAdam, K., Cuff, P., Mancher, M., Busta, E.R. (2017). Integrating Clinical Research into Epidemic Response. Washington, D.C.: National Academies Press. https://doi.org/10.17226/24739.
^,^ 4Keusch, G.T., McAdam, K.P.W.J. (2017). Clinical trials during epidemics. Lancet. 389(10088), 2455‐2457. https://doi.org/10.1016/S0140-6736(17)31602-1. In addition to EVD, many diseases without new drugs and/or vaccines are listed in the reports published by the World Health Organization (WHO),5World Health Organization. (2018). A research and development Blueprint for action to prevent epidemics. Retrieved January 20, 2018, from https://www.who.int/blueprint/en/. the Global Health Security Agenda (GHSA),6Global Health Security Agenda. (2014). Action Packages. Retrieved April 26, 2019 from https://www.ghsagenda.org/packages. and the World Bank‐sponsored International Vaccine Task Force.7Academy of Medical Sciences. (2018). Money and Microbes : Strengthening Clinical Research Capacity to Prevent Epidemics (English). Washington, D.C.: Publisher. https://doi.org/10.1126/science.301.5637.1182b.


The Clinical Research Initiative for Global Health (CRIGH)8Clinical Research Initiative for Global Health. (2019). Overview. Retrieved July 24, 2019 from https://crigh.org/://crigh.org/
 was launched in 2017 corresponding to the Organization for Economic Cooperation and Development (OECD) recommendations9OECD Global Science Forum. (2011). OECD Global Science Forum Facilitating International Cooperation in Non‐Commercial Clinical Trials Facilitating International Cooperation in Non‐Commercial Clinical Trials Organisation for Economic Co‐Operation and Development. Global Science Forum. for better global governance of international non‐commercial clinical research. CRIGH will encourage international cooperation to rapidly and efficiently respond to global health challenges which are mentioned above.

One of the major challenges is the need for multiple ethics reviews by institutional review boards (IRB) or research ethics committees (REC).10Forjuoh, S.N. (2015). Challenges Associated with Multi‐institutional Multi‐site Clinical Trial Collaborations: Lessons from a Diabetes Self‐Management Interventions Study in Primary Care. Journal of Clinical Trials. 05(03). https://doi.org/10.4172/2167-0870.1000219. International clinical trials are even more complex than single‐country studies due to the diversity of legal and ethical frameworks. Researchers and sponsors who conduct an international and multi‐site trial face two problems with ethics reviews: the differences between the countries and duplicate reviews within one country.

Information on the current ethics review system in the EU11Druml, C., Wolzt, M., Pleiner, J., Singer, E.A. (2009). Research ethics committees in Europe: Trials and tribulations. Intensive Care Medicine. 35(9), 1636‐1640. https://doi.org/10.1007/s00134-009-1544-y

^,^ 12Veerus, P., Lexchin, J., Hemminki, E. (2014). Legislative regulation and ethical governance of medical research in different European Union countries. Journal of Medical Ethics. 40(6), 409‐413. https://doi.org/10.1136/medethics-2012-101282. and USA13Grady, C. (2015). Institutional review boards purpose and challenges. Chest. 148(5), 1148‐1155. https://doi.org/10.1378/chest.15-0706. are widely available, however, there is limited information on ethics review systems for countries in other areas. Therefore, the CRIGH Research Bioethics project, co‐chaired by Council on Health Research for Development (COHRED), National Cancer Center Japan, and National Institutes of Health, conducted a cross‐sectional survey to understand the differences of ethics review systems and how these could present obstacles for promoting international collaborative trials by non‐profit, academic organizations, and to formulate recommendations to overcome these obstacles to facilitate ethical international collaborative health research and clinical trials.

We developed the survey items referring to four international standards: the EU Clinical Trial Directive (Directive 2001/20/EC),14European Commission. (2019). Directive 2001/20/EC. Retrieved April 26, 2019 from https://ec.europa.eu/health/sites/health/files/files/eudralex/vol-1/dir_2001_20/dir_2001_20_en.pdf
 the International Council for Harmonisation of Technical Requirements for Pharmaceuticals for Human Use (ICH) –Guideline for Good Clinical Practice (GCP),15ICH Harmonised Tripartite Guideline. (1996). Guideline For Good Clinical Practice. Retrieved April 26, 2019 from https://www.ich.org/fileadmin/Public_Web_Site/ICH_Products/Guidelines/Efficacy/E6/E6_R1_Guideline.pdf. Standards and operational guidance for ethics review of health‐related research with human participants published by WHO,16World Health Organization. (2011). Standards and Operational Guidance for Ethics Review of Health‐Related Research with Human Participants.; 2011. Retrieved April 26, 2019 from https://www.ncbi.nlm.nih.gov/books/NBK310666/pdf/Bookshelf_NBK310666.pdf. and 45 CFR part 46.1745 CFR 46. Retrieved April 26, 2019 from https://www.govinfo.gov/content/pkg/CFR-2016-title45-vol1/pdf/CFR-2016-title45-vol1-part46.pdf. In addition, the information from COHRED’s routine assessment of RECs using RHInnO *Ethics* 18RHInnO Ethics. Retrieved April 26, 2019 from http://www.rhinno.net. was used for the development of the questionnaire. The questionnaire contained 17 items regarding national ethics review system.

We recruited experts of ethics review system in each country by snowball sampling in Latin America, Oceania, and South East Asia. In African countries, COHRED used its network of research ethics committees to recruit national experts. The experts included chairs of IRBs/RECs, committee members, and a clinical researcher. The survey questionnaire was sent via e‐mail or Google Forms to the experts in 41 countries. Data collection periods were from November 2017 to June 2018.

We had 31 responses (response rate = 75.7%): 17 in Africa, 9 in Asia, 4 in Latin America, and one from Australia.

The legal basis of IRB/REC and the composition of committee members were similarly regulated; 81% (n=25/31) had a national requirement to establish IRB/RECs. Regarding the composition of committee members, 90% (n=28/31) required more than five members including at least one non‐scientific member in a committee, and 97% (n=30/31) required Conflict Of Interest management of committee members.

**Figure 1:**
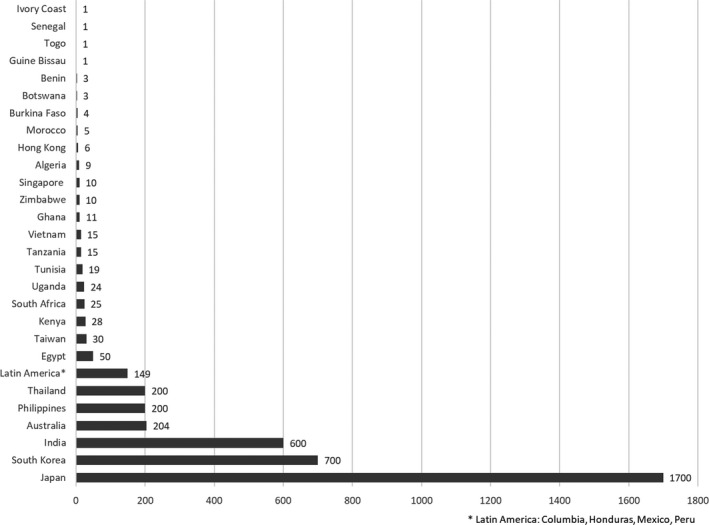
Number of IRB/RECs

There was substantial variation in the number of IRB/RECs (Figure 1), in the use of a single opinion approach, national IRB/REC accreditation, availability of a review timeframe, transparency of IRB/REC’s decisions, and training for committee members (Table 1).

**Table 1:** Variation of the requirements of laws or guidelines for the ethics review system in each area

Survey items				Countries (N=31)			
Requirements by laws or guidelines	Responses	%	n	Africa	Asia	Latin America	Oceania
Single IRB/REC opinion for multi‐site clinical trial within a country	Mandate	19	6	6	0	0	0
	Possible	23	7	2	3	1	1
	Impossible	29	9	3	3	3	0
	No requirement	23	7	4	3	0	0
	Others	6	2	2	0	0	0
National IRB/REC accreditation system	Yes	32	10	3	4	2	1
	No	68	21	14	5	2	0
Review timeframe: maximum 60 days	Yes	48	15	8	5	1	1
	Yes (other timeframe)	13	4	2	1	1	0
	No	39	12	7	3	2	0
Transparency of IRB/REC's decision*	Yes	61	19	10	7	2	0
	No	39	12	7	2	2	1
Training for committee members	Yes	74	23	10	8	4	1
	No	23	7	6	1	0	0
	N. A.	3	1	1	‐	‐	‐

* The definition of "transparency" was that IRB/REC should make the decisions publicly available, excluding confidential information.

Figure 1 shows the number of IRB/RECs in the countries whose experts responded to our survey. The number varied from one to 1700.

Our survey showed the differences in characteristics of ethics review systems, some being potential hurdles for conducting international and multi‐site trials: multiple ethics approvals for multi‐site trials within a country, lack of trained committee members to properly review the study, or different timeframes for the review. We can make four recommendations for accelerating and improving ethics review of international, multi‐site trials based on our survey results.

First, countries should strive to adopt “single opinion” for multi‐site clinical trials. A “single opinion” approach to multi‐site trial review within a country will reduce duplicate reviews and delays caused by the need to re‐review. Duplicate reviews within a country are a huge hurdle to promote an international and multi‐site trial. The EU has already mandated “single opinion” for multisite clinical trials carried out in more than one member state since 2001 based on its Clinical Trials Directive.19European Commission, op. cit. note 14. The USA has also planned to mandate single IRB review for multi‐site trial within the U.S. National Institutes of Health (NIH) system.20Gordon, V., Culp, M., Wolinetz, C. (2017). Final NIH Policy on the Use of a Single Institutional Review Board for Multisite Research. Clinical and Translational Science. 10(3), 130‐132. https://doi.org/10.1111/cts.12447. However, only 13 countries mandate a “single opinion” for a multi‐site trial within their national borders. One challenge is that a single IRB cannot review the local context of each institution included as a research site. Another challenge could be “IRB shopping,” because researchers can choose IRBs for the review, and they can submit protocols to multiple IRBs until one is found that will approve the protocol. Despite these challenges, “single opinion” would be still beneficial for promoting international and multi‐site trials. Countries should at least provide an option for researchers to choose a “single opinion” approach for a multi‐site trial within one country.

Second, national requirements should include training and continuing education in research ethics for IRB/RECs members. The training of IRB/REC members could improve the protocol review, ensuring response in an adequate time and standardizing its quality. However, not all surveyed countries required training for IRB/REC members. A potential reason may be insufficient availability of educational tools and in multiple languages. Even if a country requires training for IRB/REC members, they cannot have effective education without resources. E‐learning approaches could be effective in countries with a large number of IRB/RECs and adequate internet infrastructure, and on‐site education could be implemented in countries with a small number of committees or low internet access. We need further discussion on the development of universal training tools in several languages and their availability through online platforms. Among its future activities, our group aims to assess the landscape of available tools.

In addition to learning core competencies, IRB/REC members need to be up‐to‐date with changes in local regulation and their impact on ethics review (e.g. the General Data Protection Regulation in the EU). Moreover, we should consider streamlining the number of committees within a country to effectively and swiftly implement standardized education since countries with hundreds of IRB/REC represent a challenge to successful training.

Third, national requirements should include an adequate timeframe for the ethics review process. It is essential to establish a reasonable timeframe for the trial review, because ethics review in a country which has no timeframe requirement would cause a bottleneck for promoting an international multi‐site trial. Although the timeframe may depend on the type of review,21World Health Organization. (2018). Ethics Review Committee: review process. Retrieved December 25, 2018 from https://www.who.int/ethics/review-committee/review_process/en/
 the IRB/REC human resources and their experience, and the internal processes within each country, setting a 60‐day maximum provides an estimate for investigators, who could than plan and prepare accordingly before the international trial starts. We need to set minimal timeframe of ethics review to harmonize the starting point of international trials.

Fourth, technological innovations such as web‐based ethics review management and expert decision support platforms including RHInnO *Ethics* 22RHInnO Ethics, op. cit. note 18. also can provide solutions for the issues above, especially in the countries with under‐resourced IRBs/RECs and research systems. Many countries in Africa have implemented the RHInnO *Ethics* platform with support from the European and Developing Countries Clinical Trial Partnership (EDCTP), research funders, and international non‐profits. By providing real‐time access to a virtual REC Administrator qualified to manage complex clinical research at the level required by, for example, the US Department of Health and Human Services, the RHInnO *Ethics* platform can provide the ability to conduct high level efficient review in time to virtually any REC Administrator23Kasule, M., Wassenaar, D.R., IJsselmuiden, C., Mokgatla, B. (2016). Silent Voices: Current and Future Roles of African Research Ethics Committee Administrators. IRB Ethics and Human Research. 38(1), 13‐19. in Africa that has access to internet, and speed up clinical research substantially.24Mokgatla, B., Bahati, P., IJsselmuiden, C.I. (2017). Enhancing the Efficiency and Quality of African Research Ethics Review Processes – Through an Automated Review Platform. Journal of AIDS Clinical Research. 8(2), 2. https://doi.org/10.4172/2155-6113.1000658.


While we realize that working globally entails working with and respecting national autonomy, especially in the ethics of research, we also want to emphasize that it is time to harmonize ethics review processes for international multi‐site trials as an effective and low‐cost manner to achieve global health.

